# Autoreactive lymphocytes in multiple sclerosis: Pathogenesis and treatment target

**DOI:** 10.3389/fimmu.2022.996469

**Published:** 2022-09-23

**Authors:** Rongzeng Liu, Shushu Du, Lili Zhao, Sahil Jain, Kritika Sahay, Albert Rizvanov, Vera Lezhnyova, Timur Khaibullin, Ekaterina Martynova, Svetlana Khaiboullina, Manoj Baranwal

**Affiliations:** ^1^ Department of Immunology, School of Basic Medical Sciences, Henan University of Science and Technology, Luoyang, China; ^2^ Department of Biochemistry and Molecular Biology, Faculty of Life Sciences, Tel-Aviv University, Tel-Aviv, Israel; ^3^ Department of Biotechnology, Thapar Institute of Engineering and Technology, Patiala, India; ^4^ Gene and cell Department, Kazan Federal University, Kazan, Russia; ^5^ Neurological Department, Republican Clinical Neurological Center, Kazan, Russia

**Keywords:** T lymphocytes, B lymphocytes, biomarkers, immune pathogenesis, CNS, multiple sclerosis, therapy

## Abstract

Multiple sclerosis (MS) is a chronic inflammatory disease of the central nervous system (CNS) characterized by destruction of the myelin sheath structure. The loss of myelin leads to damage of a neuron’s axon and cell body, which is identified as brain lesions on magnetic resonance image (MRI). The pathogenesis of MS remains largely unknown. However, immune mechanisms, especially those linked to the aberrant lymphocyte activity, are mainly responsible for neuronal damage. Th1 and Th17 populations of lymphocytes were primarily associated with MS pathogenesis. These lymphocytes are essential for differentiation of encephalitogenic CD8^+^ T cell and Th17 lymphocyte crossing the blood brain barrier and targeting myelin sheath in the CNS. B-lymphocytes could also contribute to MS pathogenesis by producing anti-myelin basic protein antibodies. In later studies, aberrant function of Treg and Th9 cells was identified as contributing to MS. This review summarizes the aberrant function and count of lymphocyte, and the contributions of these cell to the mechanisms of MS. Additionally, we have outlined the novel MS therapeutics aimed to amend the aberrant function or counts of these lymphocytes.

## Introduction

Multiple sclerosis (MS) is a chronic inflammatory disease of the central nervous system (CNS) characterized by disruption of myelin sheath integrity and damage to axons and bodies of neurons (1). Initially, this destruction is microscopic in size, which accumulates over the years, presenting with CNS lesions, which could appear disseminated temporary and spatially. Clinical manifestation of MS has a wide range of neurological symptoms ranging from a loss of sensation to impaired muscle function, visual loss, cognitive dysfunction, etc. (2). The MS diagnostic criteria reflect a pattern of CNS lesion development by incorporating the signs of disseminated CNS damage identified with magnetic resonance imaging (MRI) and clinical symptoms (3, 4). Although diagnosed in all age groups, most MS patients are between 20 and 40 years old (5–8). Among them, there is a higher prevalence of MS in women as compared to men (8).

Pathogenesis of autoimmune disease is explained by a loss of immune tolerance to self-proteins due to a combination of genetic susceptibility and environmental provocation. This could result in the generation of autoreactive T and B cells. This review summarizes the latest advances in our understanding of the autoreactive lymphocyte populations that are identified as playing role in the pathogenesis of MS. The cells recently found to be affected in MS include GM-CSF-secreting effector T cells (9), DR2a and DR2b-derived self-peptides specific CD4+ T cell clones (10), glutamate producing Th17 cells (11), GM-CSF+ CXCR4+T cells (12), CD4+CD25hiCD127lowFOXP3+ Tregs (13), MBP-specific CD8+ T cells (14), circulating CD8+ CD20+ T memory cells (specific to myelin) (15) and regulatory B cells (Bregs) producing IL-10 and IL-35 (16). The autoreactive CD4+ T cells cross-reacting with DR2a and DR2b-derived self-peptides shown as a contributing to MS pathogenesis (10). The glutamate producing Th17 cells could cause damage to oligodendrocytes, exposing myelin antigens as a target to autoreactive lymphocytes (11). GM-CSF+ CXCR4+T cells express significantly higher amounts of VLA-4, a CXCR4 ligand, associated with an increase in lymphocyte trafficking to the CNS (17). Studies have shown that the MBP-specific CD8+ T cells could exacerbate brain inflammation (18). Circulating CD8+ CD20+ T memory cells were found with up-regulation of activation markers, pro-inflammatory cytokines, and adhesion molecules, indicating that they could have a high pathogenic potential (19).

About 2.8 million cases of MS were diagnosed worldwide in 2020 (7). Interestingly, a greater burden of MS was reported in countries with high socio-economic status (20). This observation is supported by the high-risk of MS in Northern Europe and North America, where ≥100 cases are diagnosed per 100,000 population (20, 21). A lower risk of MS is found in Africa and Asia, where the prevalence is <30 cases per 100,000 population (22–24). An analysis of MS frequency led to identification of three zones of global MS prevalence: high (30-80 cases per 100,000 population), medium (5-25 cases per 100,000 population) and low (<5 cases per 100,000 population) (25). An updated five-zone scale of MS prevalence was proposed by Wade: very high (170–350 cases per 100,000 population), high (70–170 cases per 100,000 population), medium (38–70 cases per 100,000 population), low (13–38 cases per 100,000 population), and very low (0–13 cases per 100,000 population) (26). The very high and high prevalence zones of MS were reported in North America and Northern Europe, while Asia and Africa were in the lower zone (27). It appears that populations with European ancestry (28) are at a higher risk, due to higher MS prevalence reported in Australia and New Zealand (27). This assumption is also supported by finding higher MS prevalence in Russia (30-<60 cases per 100,000 population) as compared to that in China (0-<30 cases per 100,000 population) (27). Within Russia, the incidence rate of MS is also higher in the North-West regions, where the population is predominantly of European ancestry (29). In China, MS prevalence remains low, although an increasing trend was recently reported (30, 31). It also appears that residence sites are one of the risk factors of MS, as more MS cases were diagnosed at high latitude and high altitude locations (31).

## Risk factors of MS

The pathogenesis of MS remains largely unknown mainly due to the limited understanding of the disease ethology. Multiple risk factors of MS were identified representing unrelated elements such as latitude, serum vitamin D (vitD) levels, genetics and virus infection (32). However, neither one of these factors was recognized as the single cause of the disease suggesting that the pathogenesis of the disease is multifactorial.

### Genetic and epigenetic risk factors

The role of the genetics in MS pathogenesis is widely recognized and supported by a large body of evidence. This includes a higher frequency of MS found in siblings (33–35) as well as in individuals closely related to MS probands (36). Also finding the link between Human leukocyte Antigen (HLA) DRB1*1501 haplotype and MS in Northern European populations supported this assumption (37). A separate subset of haplotypes, the HLA-DRB1*0301, HLA-DRB1*0405 and HLA-DRB1*1303 haplotypes, is found in MS from Mediterranean region (38). Similarly, the association between HLA-DRB1 was demonstrated in Russian and Chinese MS patients (39, 40).

Epigenetic modifications, which includes histones and DNA post-translational modification, could represent the environment induced modification of gene expression (41). These epigenetic modifications were also implemented to play role in pathogenesis of MS (42). Supporting this assumption was the study demonstrating differentially methylated region (DMR) composed of eight hypomethylated CPGs in HLA-DRB1 in MS (43). This data emphasising the contribution of hypermethylation of ring finger protein 39 (rnf39) at an independent MHC site to MS pathogenesis. In a recent study, the DMR hypomethylation in the HLA-DRB1*15:01 locus was found in the blood cells of MS patients. This study demonstrated that the hypomethylation of this MS susceptibility gene locus can induce the high expression of HLA-DRB1, found in these patients (44). Additionally, a significant hypermethylation of Foxp3 promoter was reported in EAE mice (45). It was demonstrated that expression of FOXP3 gene in EAE is significantly reduced by methylation of conserved non-coding sequence 2 (CNS2).

### Latitude and VitD serum level as risk factors of MS

Other extensively documented risk factors are latitude and serum VitD levels. A latitudinal gradient with an increased south to north prevalence of MS was documented in Northern America and Northern Europe (46). Similar patterns of higher number of MS cases in the northern as compared to southern regions within the same country is also reported (47, 48). Latitudinal gradient with lower number of cases in the south regions is found in Russia and China (29, 31). The latitude dependent risk of MS could be explained by the lesser sunlight exposure in the north as compared to south regions (32). This lesser sun exposure could also explain the commonly found lower VitD serum levels in MS patients (49). The effect of VitD levels was explained by decreased intensity of ultraviolet B (UVB) radiation in northern latitudes (50). In addition to regulation of VitD synthesis, UVB could ameliorate symptoms of MS by inhibiting production of pro-inflammatory cytokines (51).

### Virus infection as risk factor of MS

The role of viral infection in pathogenesis of MS was suggested in multiple studies (52–54). It is based on the epidemiological evidence of higher risk of MS in individuals infected with Epstein-Barr virus (EBV) and diagnosed with infectious mononucleosis (52). In a recent study, Bjornevik et al. presented strong evidence of the association between EBV infection and MS (53). This study also identified EBV as a potential infectious agent causing MS. The role of this virus in MS pathogenesis is also supported by elevated anti-EBV serum antibody titers (55) and by detection of EBV in MS lesions (56, 57). The mechanisms of EBV causing MS remains largely unknown. It is believed that EBV could inhibit the production of antiviral cytokines and proteins, and interfere with the antigen processing and presentation (58). Production of the autoreactive immune cells responsible for an aberrant self-targeting immune response is also suggested (59). This assumption is supported by findings of antibodies cross reacting with myelin basic protein (MBP) and EBV latent membrane protein 1 (LMP1) (60). This cross reactivity was explained by the homology between MBP and LMP1. The role of LMP1 in MS pathogenesis was supported by the demonstration that the exposure to this virus protein could induce myelin-reactive antibodies *in vivo* (61).

### Gut microbiome

The gut microbiome contributes to the activation and development of innate and adaptive immune responses (62). The imbalance in the interaction between microbiota and immune cells could contribute to the pathogenesis of immune-mediated disorders, such as MS. Supporting this assumption are multiple studies demonstrating changes in the microbiome in MS (63–66). These changes in the microbiome were linked to MS activity, such as the risk of relapse and detection of new lesions (67). Alterations in the gut microbiome were demonstrated in the different forms of MS, where the short-chain fatty acids producing bacteria reduced in RRMS, while bacteria associated with excessive DNA oxidation were found in SPMS (65).

Studies have demonstrated that changes in the gut microbiome could affect lymphocyte activation and differentiation. It was demonstrated that MS gut microbiota could inhibit the interaction between T-cell C-C chemokine receptor type 9 (CCR9) and its ligand chemokine (CCL25) leading to a reduction of CCR9+ CD4+ T cell counts in circulation (68). A correlation has been found between a high frequency of Th17 and increased Firmicutes/Bacteroidetes ratio (69) which was evident by an increased *Streptococcus* and decreased *Prevotella* species number. The authors state that these patients had higher MS activity. In another study, a reduced number of Clostridia species, producing short-chain fatty acids was found in PPMS (70). Using EAE model of MS, the therapeutic potency of short-chain fatty acids was demonstrated which was linked to the development of Tregs (71). Dysbacteriosis characterized by increased *Methanobrevibacter* and *Akkermansia* species combined with decreased *Butyricimonas* species was reported in MS (70). Supporting the role of *Methanobrevibacter* in MS pathogenesis was finding the reduced duration of relapse in patients with higher of this species (65). Also*, Akkermansia* species could contribute to MS pathogenesis by promoting inflammatory response by blood leukocytes (72).

These data suggest that MS is characterized by a change in gut microbiome, which could contribute to a reduction of Tregs, while promoting the development of pathogenic Th17 lymphocytes. Also, dysbacteriosis could support inflammation and facilitate the differentiation of lymphocytes with proinflammatory activity.

### Cigarette smoking

Smoking was identified as an MS risk factor in many studies (73–75). Manouchehrinia *et al*’s study confirmed smoking as a risk factor by demonstrating that “ever-smokers” had a 41% higher chance of MS diagnosis as compared to “never smokers” (75). Smoking could affect the immune system function by inducing oxidative stress, the release of pro-inflammatory cytokines, and increasing nitric oxide (76–78). Cigarette smoking could contribute to MS pathogenesis by affecting the differentiation and activation of the different lymphocyte populations. It was demonstrated that smoke could trigger the development of auto-aggressive T cells, cross-reactive with CNS antigens in MS (79). Proinflammatory T cells producing IL-17 were increased in MS smokers (80). A study by Hedstrom et al. had shown a 41% risk of having MS in subjects having HLA-DRB1*15 without HLA-A*02 (81). Smoking could also have epigenetic mechanisms contributing to MS pathogenesis by affecting DNA methylation (82).

These data indicate multiple mechanisms of cigarette smoking contributing to MS pathogenesis. It could be suggested that smoking contributes to MS pathogenesis by the combined effect of activation of pro-inflammatory leukocytes, epigenetic modification, and genetic markers of predisposition to the disease.

### Sex of the patient

The sex of the patient contributes to the risk of MS, due to a reported 3:1 female to male ratio (83). It appears that puberty, early onset of menarche in particular contributes to the risk of MS diagnosis (84). It remains unclear whether this is a result of the rising level of sex hormones. However, evidence suggests that female sex hormones could contribute to the progression of the disease. This assumption is supported by reports showing amelioration of MS symptoms during pregnancy (85). Also, a reduction of disability levels during pregnancy in MS was reported (86), although, lack of long-term effect on disability scores was also documented in other studies (85, 87).

Although the sex of the patient appears to be a risk factor, there is still a lack of strong evidence establishing sex hormones as contributing to onset of MS pathogenesis. Therefore, it could be suggested that sex could contribute to MS when combined with other risk factors. Supporting this assumption is the study by Irizar et al. demonstrating the association between female sex and HLA-DRB1*15:01 haplotype (88). A combination of HLA-DRB1*15:01, an established MS risk factor, with female sex was also demonstrated by Chao et al. (89). The authors also found that this haplotype is more likely to be transmitted from a mother to a girl rather than to a boy.

Female sex hormones were shown to modulate the immune response by promoting the development of Tregs, reducing Th1 and Th17 cells activity, as well as supporting Th2 lymphocytes differentiation (90–92). The anti-inflammatory effect of estrogens was also confirmed using EAE, an MS model (93, 94).

### Obesity

Childhood obesity was reported as a risk factor for MS (95, 96). It appears that having a high body mass index (BMI) in childhood could increase the probability of MS diagnosis (96). The odds ratio for MS diagnosis increased as BMI increased reaching 3.76 in extremely obese individuals (65). Interestingly, obesity was significantly associated with MS diagnosis in girls, while it was not found in boys (95). In another study, obesity was demonstrated as a risk factor during adolescence (97), while it had a limited contribution in children. These differences could be attributed to using different approaches, BMI or body silhouettes, to assess obesity (96, 98). Still, childhood obesity is believed to contribute to MS diagnosis, as Ascherio et al. suggested reduction of childhood obesity could reduce MS diagnosis by15% (99).

Chronic inflammation is commonly found in obese patients (100). Activation of inflammatory cytokines such as IFN-γ and TNF-α was demonstrated in obese individuals (101). This inflammation could be promoted by a disturbed gut microbiome, which is often found in this group of patients (102). This gut dysbiosis could shift the Treg/Th17 ratio toward pathogenic Th17 population (103), which has been linked to pathogenesis of MS (104). Association between obesity and low levels of vitD, another risk factor of MS was found (46, 105, 106). These data suggest that the higher risk of MS could be the combined result of many risk factors, such as inflammation, Th17 activation, and low vitD levels induced by obesity.

## Pathogenesis of MS

Several hypotheses were developed to explain the pathogenesis of MS, including the role of viral infection and genetic predisposition, which are studied more extensively. Although the trigger of MS remains unknown, the consensus is that pathogenesis is based on the activation of auto-aggression against myelin proteins, causing defects in the CNS structure. These myelin proteins form a multilamellar myelin sheath around the axons and neuron cell bodies. The sheath is composed of several proteins such as MBP, myelin-associated glycoprotein (MAG) and proteolipid protein (107). Also, a myelin oligodendrocyte glycoprotein (MOG) is located on the surface of the myelin sheath (108) and functions as an adhesive receptor as well as connects neighbouring myelinated fibers (109).

Abnormalities in immune mechanisms were suggested as protagonists in pathogenies of MS. This assumption is based on finding of the reduced number and activity of circulating T-regulatory (Tregs) cells, which correlated with an exacerbation of the disease symptoms (110, 111). Tregs counts were shown to be reduced during relapse, and restored during remission in MS patients (111). The role of immune mechanisms in the pathogenesis of MS was also confirmed by histological studies, indicating the presence of leukocyte infiltrate in the plaques within the CNS (112, 113). One of the most substantial pieces of evidence supporting the immune mechanisms of the disease is the detection of antibodies and leukocyte clones specific to brain antigens, among which the MBP appears the most immunogenic (114, 115). However, the mechanism of the autoimmune response activation in MS remains unclear.

There is substantial clinical and experimental data identifying MOG as a target for auto-reactive leukocytes in MS. This assumption is supported by detection of anti-MOG antibodies in some MS patients (116, 117), indicating the development of auto-reactive B cells. An increased count of MOG-reactive T cells in blood and cerebrospinal fluid (CSF) of MS patients was reported (117). The role of MOG in MS pathogenesis was also confirmed in the experimental autoimmune encephalitis (EAE) mouse model, where this glycoprotein was shown as inducing an encephalitogenic T cell response and antibody-mediated demyelination (118). Augmentation of demyelination in EAE by monoclonal antibodies directed against MOG further supports the pathogenic role of this protein in brain demyelination (119). Some of the strongest evidence for MOG contribution to MS pathogenesis comes from the clinical study by Berger et al. with detection of anti-MOG and anti-MBP IgM, predicting the conversion of a clinically isolated syndrome, a fits episode of neurological symptoms prior the disease, to MS (116).

In addition to MOG, studies have demonstrated the presence of circulating antibodies against MBP in MS patients (114, 115). Accumulation of T- lymphocytes specific for MBP was also demonstrated in the CNS (120) and CSF (18). Additionally, studies showed prolonged circulation of MBP specific lymphocytes in the blood of MS patients (18). The role of MBP-specific lymphocytes in the pathogenesis of MS was demonstrated in the EAE model, where the transfer of lymphocytes specific for this myelin protein induced symptoms similar to those of MS (121). Phenotype studies have identified a population of myelin-specific lymphocytes as effector cells or memory cells (122, 123).

These auto-aggressive T lymphocytes could cross the blood-brain barrier (BBB) and initiate myelin destruction in the CNS. BBB is formed by tightly connected endothelial cells located at the luminal surface of the brain blood vessels (124). In addition to the endothelial cells, astrocytes, a neuroglia cells, maintain the integrity of BBB (125) by locating between the endothelial cells and neurons. Astrocytes form a perivascular end-feet at the BBB controlling the movement of molecules between brain and blood (126). This barrier function of BBB was shown to be altered in many neuroinflammatory diseases, including MS (127). It is believed that the disruption of the BBB integrity is one of the critical initial steps in MS pathogenesis, which is required for auto-reactive T cells infiltration of CNS (125). The mechanism of T lymphocyte infiltration could be explained by an increased expression of adhesion molecules on BBB endothelial cells in MS (128). The role of adhesion molecules in leucocyte trans-BBB migration was also supported by their expression on inflammatory cells such as macrophages and lymphocytes in MS lesions (129, 130).

Once the BBB integrity is disrupted in MS, it could lead to CNS infiltration with activated lymphocytes, which is facilitated by an increased expression of adhesion molecules on endothelial cells, and inflammatory cytokines (131). CD4+T lymphocytes play a key role in initiating and maintaining the autoimmune response in MS (131). Studies have also shown the essential role of CD8+ T cells in maintaining the myelin damage and inflammation. It is generally accepted that cytokines secreted by Th1 cells, such as IFN-γ and TNF-β, can activate macrophages to damage oligodendrocytes, resulting in pathological myelination (132). HLA-E-restricted CD8+ Tregs induced by IFN-γ, could reduce CD4+T lymphocyte counts and induce secretion TGF-β by local cells (133). Studies have shown that inhibiting Th1 and/or Th17 cells and increasing the number of circulating Tregs could suppress the progression of MS (134). CD8+T cells rather that CD4+ lymphocytes were found more characteristic in MS plaques (135).

## MS immune response

### Autoreactive T cells in MS

**Figure 1:**
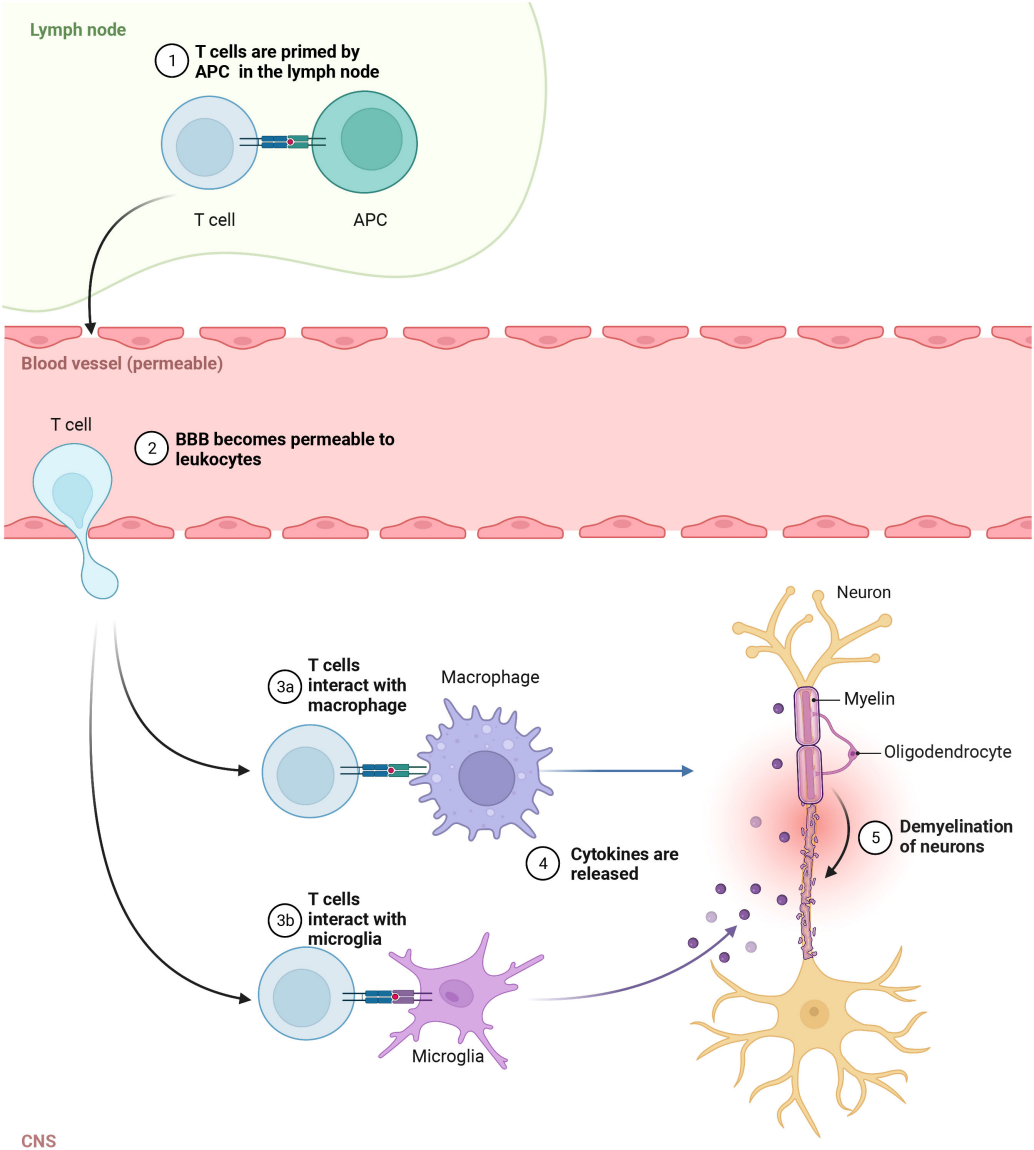
Autoreactive T cells in pathogenesis of MS. In the lymph node, T cells are primed to CNS autoantigens presented by APC, then cross the Blood brain barrier (BBB) and enter the brain. In CNS, they activate macrophages and microglia, which release cytokines promoting local inflammation and tissue injury.

MS pathogenesis is explained by leukocytes, mainly T and B lymphocytes, infiltration of the brain and spinal cord (136). It was suggested that the inflammation in the CNS selectively recruits autoreactive T cells which could target the autoantigens in brain tissue (26). It is believed that the autologous myelin reactive T cells are initially primed to CNS autoantigens in the periphery and then cross the BBB entering the brain (137). Within the brain tissue, these leukocytes could activate microglia and macrophages, promoting local inflammation (138) (**Figure 1**). It appears that the lymphocyte distribution is population specific where CD8+ T cells are mostly found at the edge, while CD4+ T cells are located deep within the lesions (139). Also, their role in the diseases’ stage was demonstrated as CD8+ T cells were frequently found in acute lesions (140). In addition to the autoreactive lymphocyte’s migration across the BBB, the reduced regulatory T cell function was shown to promote autoimmune response in MS (141). The hyperactivation of Th1 cells could lead to tissue damage and chronic inflammation (142), which is frequently found in autoimmune diseases (143). Th17 cells are known for secreting high levels of interleukin (IL) 17A, IL-17F, IL-21 and for low production of interferon γ (IFN-γ) (144). Cytokines, such as transforming growth factor β (TGF-β), IL-6, IL-1β and IL-23, are implicated in Th17 development and maintenance (145). In another study, the high frequency of Th1, Th17 and granulocyte-macrophage colony-stimulating factor (GM-CSF)-secreting effector T cells have been reported in MS (146). Together, these cells could cause the death of myelin-producing oligodendrocytes, directly damage the myelin sheath of the nerve fibers, and impact brain tissue integrity forming lesions in the CNS.

### CD4+ T cells

**Figure 2:**
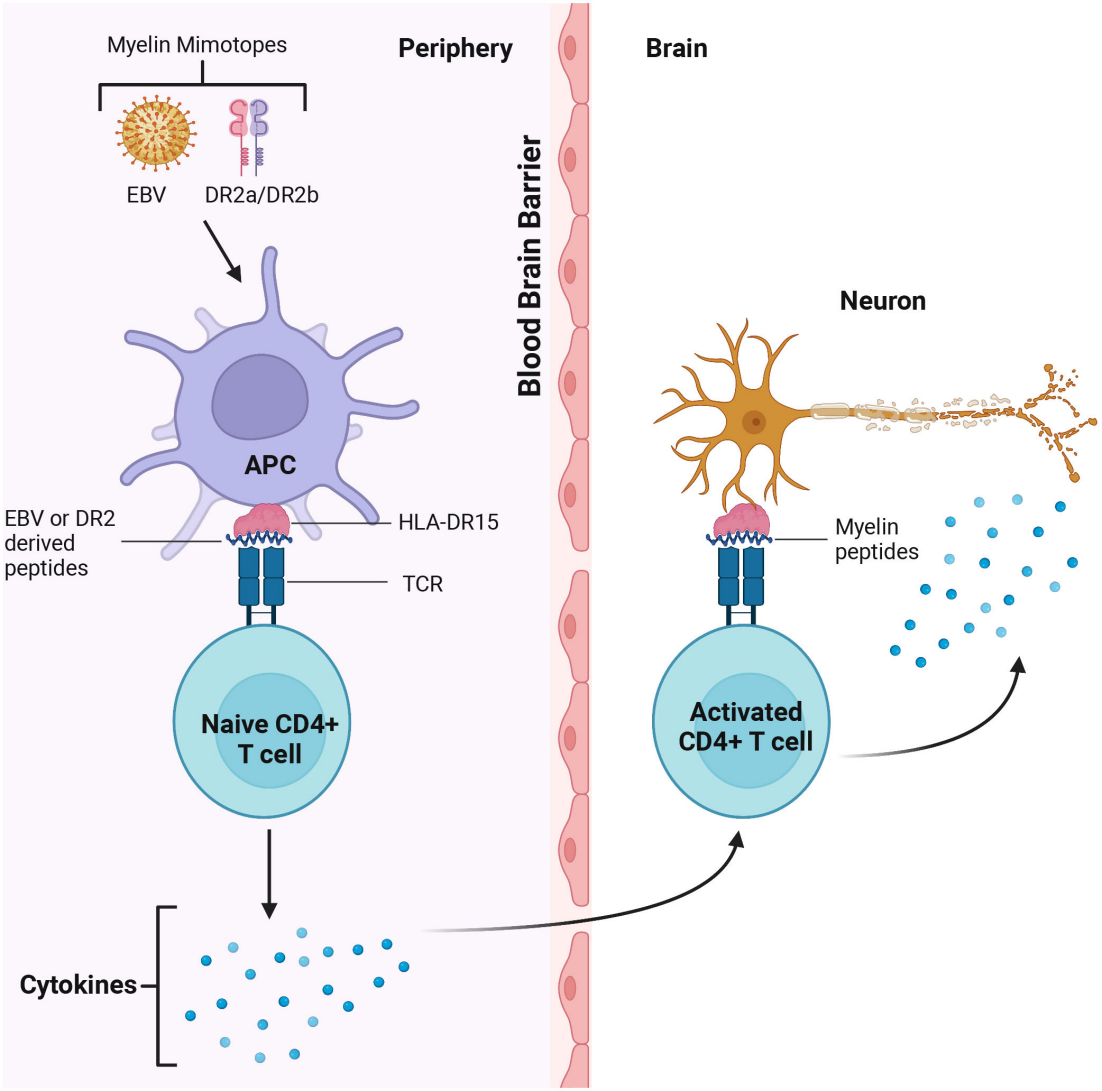
CD4+T cells cross react with Epstein Barr Virus (EBV) and DR2 derived peptides in the periphery and myelin in the brain. Myelin mimotopes could induce autoreactive CD4+ T cells by presenting EBV and DR2a/DR2b derived peptides sharing similarities with CNS self-antigens (i.e. myelin). These T cells could recognize the target antigen on microglia, leading to activation of CD4+ T cells. Activated T cells release cytokines which could trigger demyelination and axon loss, CNS inflammation and tissue injury.

The key role of CD4+ T cells was demonstrated when activated myelin-reactive CD4+ T cells were found in the blood and CSF of MS patients (12). In contrast, only non-activated myelin-reactive T cells were detected in controls. Study of CD4+ cells contribution to MS pathogenesis identified the HLA-DR15 allele as an antigen-presenting structure and epitope source to autoreactive CD4+ T cells (147). The authors identified the autoreactive CD4+ T cell clones that can cross-react with DR2a and DR2b-derived self-peptides, as well as peptides from foreign agents shown as potential cause of MS (147). CD4+ T cells could coordinate the adaptive immune response by releasing cytokines and chemokines supporting the activation of myelin-specific CD4+ T cells and their infiltration of the brain (10) (**Figure 2**).

CD4+ T cells could differentiate into distinct Th cell populations, identified by the repertoire of the surface receptors and the cytokines secreted (148). To date, several Th cell populations are recognized and characterized by secretion of the lineage-defining cytokines (149). Among these, Th1 and Th17 cell counts were shown to be increased at the onset of MS (150). Secretion of IFN-γ is characteristic for Th1 cells, which are essential to mediate T-cell immune response (151). Th17 cells are characterized by expression of retinoic acid-related orphan receptor gamma t (RORγt) as well as a signal transducer and activator of transcription 3 (STAT3) factor regulating the production of pro-inflammatory cytokines IL-17, IL-6, IL-21, IL-22, IL-23 and tumor necrosis factor α (TNF-α) (152). Another subset of lymphocytes, named Th9 producing IL-9, was shown as involved in regulation of the balance between Th17 and Tregs (153, 154). An additional population of Th lymphocytes was identified by Galli et al. which was characterized by secretion of GM-CSF and expression of CXCR4 in relapsing-remitting MS (RRMS) (146, 151).

### Th17 cells

High Th17 cell counts and elevated IL-17 mRNA transcription in the blood of MS patients were found correlated with the severity of the disease (155). Large numbers of Th17 cells were found in CSF at the early stage of MS (156). These cells were characterized by high-level expression of very late antigen-4 (VLA-4), a surface adhesion molecule for CD4+T cells, directing their migration across the BBB (156). Therefore, it was suggested that the inhibition of VLA-4 expression can reduce the CD4+ T cells migration to CNS and limit CNS inflammation (156). Th17 cells have multifactorial contribution to MS pathogenesis. These cells could produce glutamate upon contacting with oligodendrocytes in CNS (11, 157). This high glutamate level could cause damage to oligodendrocytes, exposing the myelin antigens as a target to autoreactive lymphocytes (157). Th17 could contribute to chronic CNS inflammation (151). It was shown that Th17.1 subpopulation, expressing the highest level of IL-23 receptor and granzyme B, has a greater capacity to migrate through the BBB (156). Upon stimulation with IL-23, these IL-23R+ Th17 lymphocytes could produce GM-CSF, which could change their phenotype to be more encephalitogenic and support CNS inflammation (158). This is consistent with findings in EAE model, where Th17 cells, maintained in the presence of IL-23, became cytotoxic to oligodendrocytes and neurons (156). Similarly, upregulation of GM-CSF and more encephalitogenic phenotype of Th17 was demonstrated upon exposure these cells to IL-1β (158), a proinflammatory cytokine product of inflammasome (159).

### Th9 cells

IL-9-producing CD4+ Th cells, Th9, are the most recently discovered lymphocyte subset (154). These cells are characterized by a substantial production of IL-9, as well as IL-10 and IL-21 (160). Initially identified as a sub-lineage of Th2 cells, they require IL-4 for differentiation (161). However, unlike Th2, TGF-β was shown critical for Th9 differentiation (162). The role of Th9 in the pathogenesis of MS was suggested as the IL-9 levels in CSF correlated inversely with indexes of inflammatory activity, neurodegeneration, and progression disability (163). These data suggest that Th9 product, IL-9, could have anti-inflammatory effect in MS, consequently promoting remission. Supporting this statement were findings using the EAE model. It was demonstrated that mice with EAE receiving Th9 cell had fewer lymphocyte infiltration in the meninges as compared to those receiving Th1 and Th17 cells (164). Protective role of IL-9 was also shown by using IL-9R knockout mice, which had a severe course of EAE (165). Interestingly, it appears that TGF-β could support the differentiation of Th9 as well as non-pathogenic Th17 (166). These, non-pathogenic Th17 were also shown to produce IL-9 (166). These data suggest that IL-9 produced by Th9 and non-pathogenic Th17 could be a potential therapeutic target for treatment of MS.

### GM-CSF+ CXCR4+T cells

An expanded Th cell subset was identified in CSF of RRMS patients, which was characterized by expression of GM-CSF and CXCR4 (146). CXCR4 could promote migration of lymphocyte subsets with encephalitogenic capacity across BBB in MS. Supporting the role of these cells in pathogenesis of MS was a finding in twins expressing significantly higher amounts of VLA-4, a CXCR4 ligand, associated with an increased lymphocyte trafficking to the CNS (167). GM-CSF and CXCR4 expressing cells were increased in MS as compared to other inflammatory and non-inflammatory conditions (146). The number of these cells was also reduced under the effective disease-modifying therapy by using dimethyl fumarate (DMF). These data indicate that these cells could represent a specific therapeutic target in MS. This statement was supported by the fact that knocking out GM-CSF substantially decreased the disease severity, which was associated with lower lymphocyte infiltration and inflammation in CNS (168).

### Tregs

**Figure 3:**
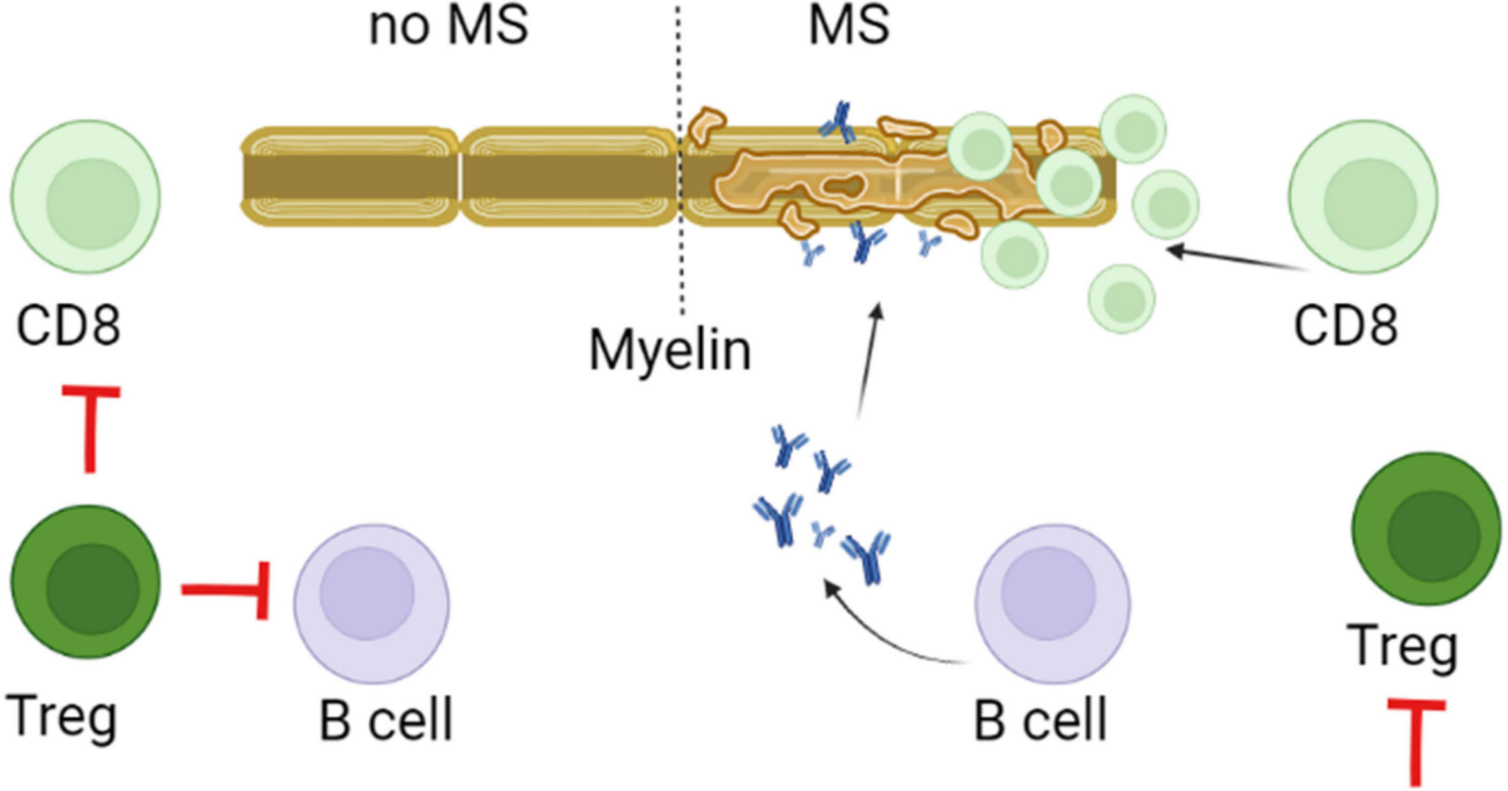
Role of Tregs in pathogenesis of MS. Tregs control of the immune homeostasis focused on preventing differentiation, proliferation and survival of autoreactive myelin targeting CD8+, Th17 and B cells. This control depends on the direct contact as well as the release of IL-10 and TGF-β by Tregs. It is believed that in MS, decreased activity and lower counts could reduce the regulatory capacity of Tregs. This could promote survival, proliferation and differentiation of myelin reactive CD8, Th17 and B cells.

Tregs have an immune-modulating function by the release of IL-10 and TGF-β (169). Multiple types of Tregs were shown to contribute the maintenance of the immune homoeostasis, by keeping autoreactive T and B cells from developing into the auto-aggressive type (**Figure 3**). In MS, the aberrant activity of effector T cells could be a result of reduced Treg function (170, 171). This assumption of insufficient Tregs function is supported by decreased counts of resting population of this lymphocytes in MS as compared to controls (172). Also, T cells appear resistant to regulation by Tregs in MS. This deficiency could lead to differentiation of pathogenic lymphocytes in MS (110, 173).Therefore, these population of T cells could be a potential target for MS (174). Support for this is found in approved disease-modifying drug, such as IFN-β for MS increased Treg cell count, which could explain the mechanism of their therapeutic efficacy (174, 175).

Since myelin-reactive T cells are found in healthy individuals, it was suggested that MS pathogenesis could be associated with insufficient immune regulation by Tregs (176). Tregs could suppress the activity of Th1 and Th17 effector cells (Teff), cytotoxic CD8+ T cells (Tc), and antigen-presenting cells (APCs) by direct contact and secretion of suppressive cytokines (177). By presenting myelin, APCs could trigger activation and differentiation of pathogenic Th1 and Th17 cells, releasing pro-inflammatory cytokines such as IFN-γ, TNF-α and IL-17 (178). The encephalitic lymphocyte activation could be suppressed by Tregs which inhibits the inflammatory response by reducing pro-inflammatory cytokine release and by promoting T cell anergy or apoptosis (177). However, the functionally defective Tregs could contribute to the pathogenesis of MS (176). There are two Tregs subpopulations, that express either CD4 or CD8 markers (179, 180). CD4+Tregs express the cytotoxic T-lymphocyte-associated protein 4 (CTLA-4) which contributes to inhibition of B and T cells proliferation and APCs function (181–183). The CD8+Tregs are characterized by the ability to suppress activation and proliferation of autoreactive CD4+ effector cells by cell-to-cell contact (184). Several immunosuppressive markers such as CD25, CTLA-4, PD-1, GITR, and transcription factors FoxP3 and Helios were found on both CD8+ and CD4+ Tregs (185). It was shown that Helios could stabilize the suppressive phenotype of both CD4+ Tregs and CD8+ Tregs, by activating the STAT5 signalling pathway (186). Interestingly, the expression of Helios in RRMS was shown to be lower as compared to controls (186). These data suggest that these Tregs in MS could have an unstable phenotype and, when migrated to CNS lesions having inflammatory milieu, they fail to continue maintaining immunoregulatory function (187).

### CD8+ T cells

Studies have shown that the MBP-specific CD8+ T cells could exacerbate brain inflammation (18, 188). The encephalitogenic effect of myelin-specific CD8+ T cells in MS could involve the FasL dependent mechanisms, especially those promoting the formation of intracerebellar lesions (188). CD8+ T cells are the primary lymphocytes found in the CNS brain lesions of MS patients and mice with EAE (18). Supporting the role of CD8+ T cells in MS pathogenesis, was a finding that the adoptive transfer of CD8+ enriched MOG-specific T cells could induce EAE in mice (189). In another study, an increased number of circulating CD8+ CD20+ T memory cells, specific to myelin antigens was found in MS as compared to control subjects. The expression of CD20 on these T cells correlated with up-regulation of activation markers, pro-inflammatory cytokines and adhesion molecules, indicating that they could have high pathogenic potential. This assumption was supported by experiments with an adoptive transfer of CD20+ T cells in EAE mice, which caused the deterioration of the brain tissue integrity and aggravation of the disease severity (190). The proportion of memory and CD20+CD8+ T cells, specific for myelin antigens, was significantly reduced following anti-CD20 treatment, indicating that the depletion of CD20+ T cells has therapeutic potential in MS (190).

### B cells

Originally, MS was believed to be a primarily T-cell mediated disease with an imbalance of pro- and anti-inflammatory cells in CNS inflammation. However, more evidence suggests that B cells are also involved in the pathogenesis of disease. Though not completely clear, but both, antibody-dependent and independent mechanisms, are now proposed as mechanisms of B-cell mediated CNS injury in MS (188). Aberrant stimulation of B cells and plasma cells are suggested in MS (191). These cells could also inflict damage by producing autoantibodies against specific myelin antigens. They could produce autoantibodies affecting the course of MS (192). In the EAE model, B cells were shown to interact with CD4+ T cells and initiate an adaptive immune response to myelin antigens. Reduced inflammation and alleviated clinical scores were shown in rats with EAE after inhibiting B cell immunity (193, 194). These data suggest that B cells could be used as therapeutic targets for MS.

The direct evidence of the role of B cells in immunity comes from histology analysis of the CNS. Lymphatic follicle-like aggregates containing B cells were shown in meninges of MS patients, similar to the secondary lymphatic tissue developed at sites of chronic inflammation (195). The role of these aggregates in MS pathogenesis is supported by finding their association with more severe neuropathology and clinical disease. These data identified the CNS location of auto-reactive B cells in MS, which is the important for our understanding of the disease pathogenesis as well as it provides the site for the potential therapeutic targeting. Significant B cell infiltration is often found along the small blood vessels at the site of myelin destruction, suggesting the continuous production of mature B cells in CNS causing local humoral immune response (196). This sustained proliferation of B cells producing anti-myelin antibodies could cause damage to the cerebral cortex and exacerbate the progression of MS. Recently, B-cell depletion with anti-CD20 monoclonal antibodies was demonstrated as highly effective for treatment of all forms of MS (197–200). The depletion of B cells suppressed the inflammation and reduced the MS relapses, suggesting that B cells play a central role in the pathogenesis of MS.

Additionally, B cells could produce cytokines contributing to MS pathogenesis. By producing IL-6, B cells could bias towards differentiation of pathogenic Th cells capable of triggering EAE (201, 202). This IL-6 derived pathogenic Th lymphocytes were linked to expression of Fc fragment of IgM receptor (FCMR) gene in Th17.1 population (203). The surface receptor FCMR was identified as unique regulator of inflammatory autoimmune responses and an attractive target for therapeutic intervention. Inhibition of FCMR activity *in vivo* at an early or late stages of EAE could prevent the disease progression (204). Additionally, B cells could produce GM-CSF, a cytokine linked to the pathogenesis of MS (145, 205).

The role of regulatory Bregs in pathogenesis of MS is extensively explored in the last two decades. Bregs can mediate immune tolerance and inhibit inflammation by producing IL-10 and IL-35 (206). IL-10 is anti-inflammatory cytokine which can suppress the development of Th1 and enhance polarization of Th2 lymphocytes (207, 208). Similar to IL-10, IL-35 has strong anti-inflammatory activity by inhibiting differentiation of pathogenic Th1 and Th17 as well as promoting development of Bregs and Tregs (209, 210). In addition to IL-10 and IL-35, Bregs could secrete TGF-β, thus, suppressing Th1 proliferation while promoting Treg cells differentiation (211). The role of Bregs in pathogenesis of MS was demonstrated in a study, where the reduction of the naïve/memory Bregs was found during a relapse (212). Further, Kim et al. demonstrated reduced counts of immature and highly regulatory Bregs in MS (213). These data support the notion that Bregs could be a novel target for MS therapy. The study has shown that treatment with alemtuzumab, an anti-CD52 monoclonal antibody, increased these lymphocyte counts, which correlated with the efficacy of MS therapy (214). Similar results were presented by Zhang et al. confirming the efficacy of alemtuzumab treatment which was linked to reconstitution of these lymphocyte subset population (215).

Studies suggest that EBV could be one of the triggers of MS (215). EBV infection also has a connection to B cells as it could establish latent infection in memory B cells (216). Infection of naïve and mature B cells with EBV could happen *via* CD21, CD35 and HLA-DR, which contribute to the viral load and the expansion of B cells (217). EBV produces latent member protein 1 (LMP1), which can promote the survival and immortalization of B cells (56). These LMP1 expressing B cells are often found in the brain of MS (56). Therefore, it was suggested that the increased prevalence and the association between EBV and MS could be a consequence of chronic B cell activation in these patients (218).

In conclusion, it appears that B cells are involved in the MS pathogenesis through (I) presenting antigenic peptides to T cells and driving the self-proliferation of brain homing T cells (possibly through memory B cells) (219), (II) producing regulatory cytokines and chemokines and contributing to differentiation of lymphocyte subpopulations and (III) serving as a site for EBV infection.

### Cytokines

Cytokines are small molecules with pleiotropic function enabling cell communication in health and in pathology. Cytokines could define the resolution of the pathological process by contributing to the recovery and development of the immune response. However, in some cases, they can support the destruction of the host tissue, promoting the aberrant immune response. The role of cytokines in pathogenesis of MS was demonstrated in multiple studies identifying molecules contributing to the development of inflammation, auto-aggressive T lymphocytes and nervous tissue damage (220–224). Cytokines could contribute to the pathogenesis of chronic inflammation in MS as well as orchestrating leukocytes trans-BBB migration.

The current paradigm of MS pathogenesis is explained by activation of Th1 and Th17 lymphocytes (225). Activation of these lymphocyte populations is managed by a specific set of cytokines. The Th1 cells differentiation is regulated by IFN-γ, IL-2 and TNF-α (150, 226). Also, activated Th1 can secrete IFN-γ and TNF-α, which maintain the inflammatory milieu and provide positive feedback to support differentiation of naïve T cells to Th1 (227). These Th1 lymphocytes assist differentiation of cytotoxic T lymphocytes (CTLs), playing a central role in myelin disintegration (228). There are vast numbers of data supporting the role of IFN-γ and TNF-α in pathogenesis of MS. These data demonstrated that IFN-γ is expressed in MS lesions (222, 223, 226, 229). Also, it appears that the overexpression of IFN-γ in the CNS could contribute to demyelination by opposing the remyelination (230). Similar to IFN-γ, increased serum and CSF levels of TNF-α in MS patients correlated with the disease severity and progression (231–233). Additionally, the exacerbation of the disease symptoms was shown to correlate with an increased serum level of this cytokine (234). Demyelination appears to depend on TNF-α expression as it correlates with the loss of myelin in grey matter at the time of diagnosis and postmortem (235).

Th17 lymphocytes differentiation is tightly regulated and requires TGF-β and IL-6 (236). Cytokines IL-21 and IL-23 support the proliferation and stabilization of Th17 lymphocyte population (145). In the absence of TGF-β, a cocktail of IL-6, IL-23 and IL-1β could control differentiation of Th17 (166). It was demonstrated that the exposure of Th17 lymphocytes to IL-23 results in conversion of non-pathogenic to pathogenic phenotype (145, 166). Increased serum level of IL-6, IL-23 and IL-1β was demonstrated in MS (145, 223, 237). Interestingly, reduced in serum, while increased in CFS was the level of TGF-β, a key regulator of Th17 differentiation (238, 239). This elevated TGF-β in CSF correlated with the disease relapse (240).

Studies have shown the Th17 lymphocyte plasticity, where they could shift to Th1 cells (238, 241). This was confirmed by finding Th17 phenotype lymphocyte population producing IL-17 as well as IFN-γ, a cytokine characteristic for Th1 cells (242). Similarly, Th17/Th1 phenotype of Th17 lymphocytes was reported by Cosmi et al. in patients with juvenile idiopathic arthritis (242). This ability of Th17 to convert to pathogenic Th1 lymphocytes was demonstrated using EAE model of MS (243). It appears that, depending on the cytokine microenvironment (243), Th17 could obtain the pathogenic features of Th1 lymphocytes, producing IFN-γ (244). The leading role in this switch is played by IL-12, IL-23 and IFN-γ (243).

Cytokines could also contribute to migration of autoreactive T and B cells across the BBB. Supporting this, TNF-α was shown to affect the distribution of tight junction and adherents junction molecules, holding the endothelial cells together and reducing BBB permeability (244). Also, a combination of TNF-α and IFN-γ could alter the architecture of the junction proteins. Additionally, these cytokines could increase the expression of adhesion molecules such as intercellular adhesion molecule 1 (ICAM), vascular cell adhesion molecule 1 (VCAM-1) and selectins, which are required for leukocytes trans-endothelial migration (245, 246). Similarly, IL-6 was shown to increase the VCAM-1 expression and leukocyte recruitment to the spinal cord. Cytokines, associated with Th17 lymphocyte activation also contribute to lymphocyte migration across the BBB. It was demonstrated that endothelial cells express IL-17R and IL-22R, promoting disruption of BBB integrity in MS patients (247). It appears that IL-17 promotes lymphocyte migration by increased expression of chemokine (C-C motif) ligand 2 (CCL2), IL-6 and chemokine (C-X-C motif) ligand 8 (CXCL8) by endothelial cells of BBB. Increased serum level of these chemokines was also found in MS patients (222).

**Figure 4:**
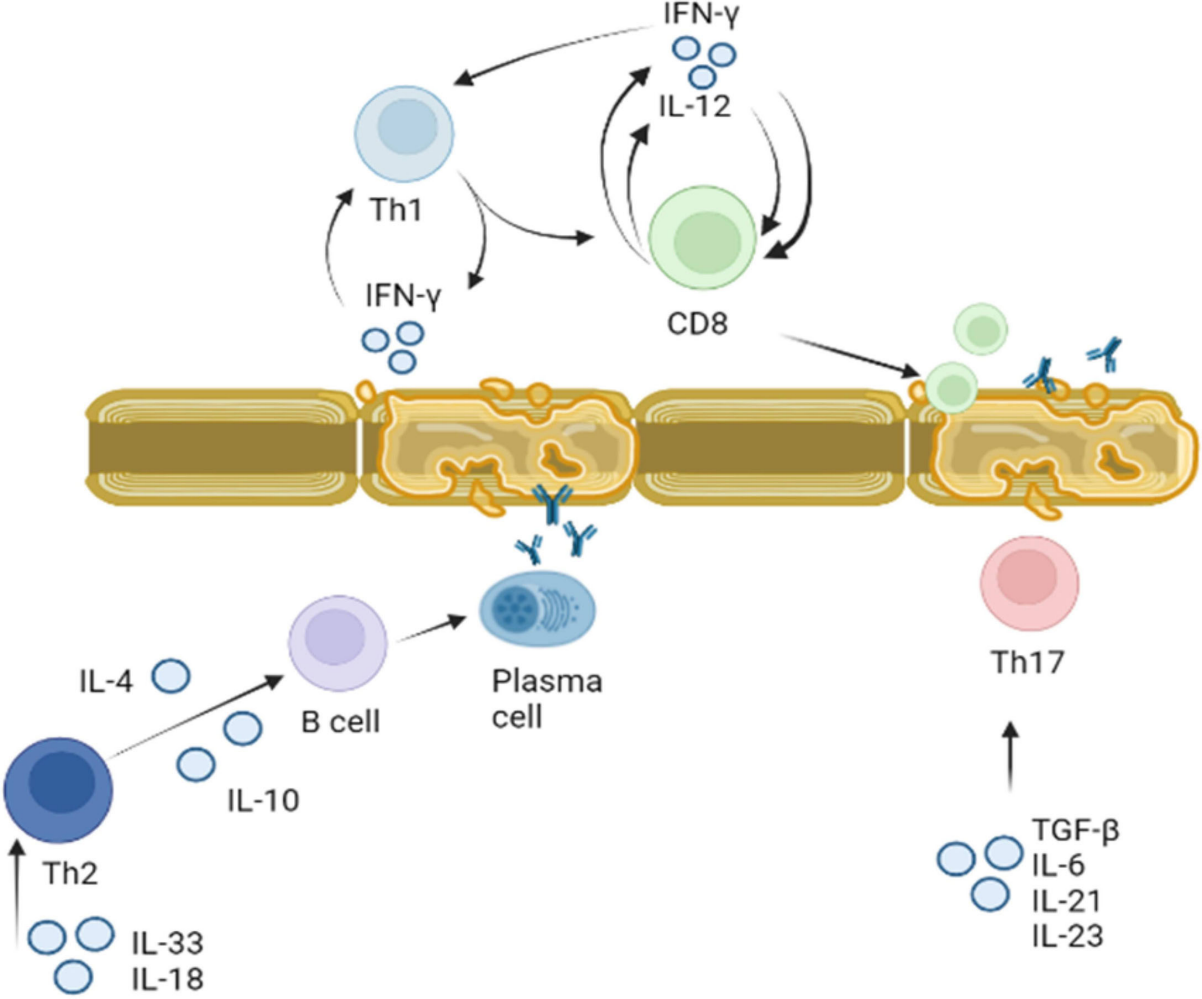
Role of cytokines in activation of T and B lymphocytes in MS. Activation of Th1 and Th2 lymphocytes was demonstrated in MS. Th1 lymphocyte differentiation is regulated by IFN-γ. Once Th1 cells are activated they produce more IFN-γ stimulating differentiation and proliferation of CD8 cells. CD8 cells also release IFN-γ, which together with Il-12 supports CD8 and Th1 proliferation. Activated myelin primed activated CD8 lymphocytes could damage myelin sheets. Activated by IL-18 and IL-33, Th2 lymphocytes secrete IL-4 and IL-10 which support proliferation and differentiation of B cells. Myelin antigen primed B cells could terminally differentiate to plasma cells producing anti-BMP antibodies targeting myelin sheets. Th17 lymphocytes proliferation and differentiation is supported by IL-6, IL-21, IL-23 and TGF-β. These activated Th17 lymphocytes could also target axon myelin sheets and contribute to the local inflammation.

These data suggest that cytokines play a role in pathogenesis of MS by: I. directing differentiation of T lymphocytes towards the auto-reactive Th1 and Th17 phenotype and II. disrupting the integrity of BBB and facilitating migration of these reactive leukocytes. Therefore, cytokines as well as autoreactive lymphocytes could be therapeutic targets for treatment of MS (**Figure 4**).

## MS treatment targeting lymphocytes

**Figure 5:**
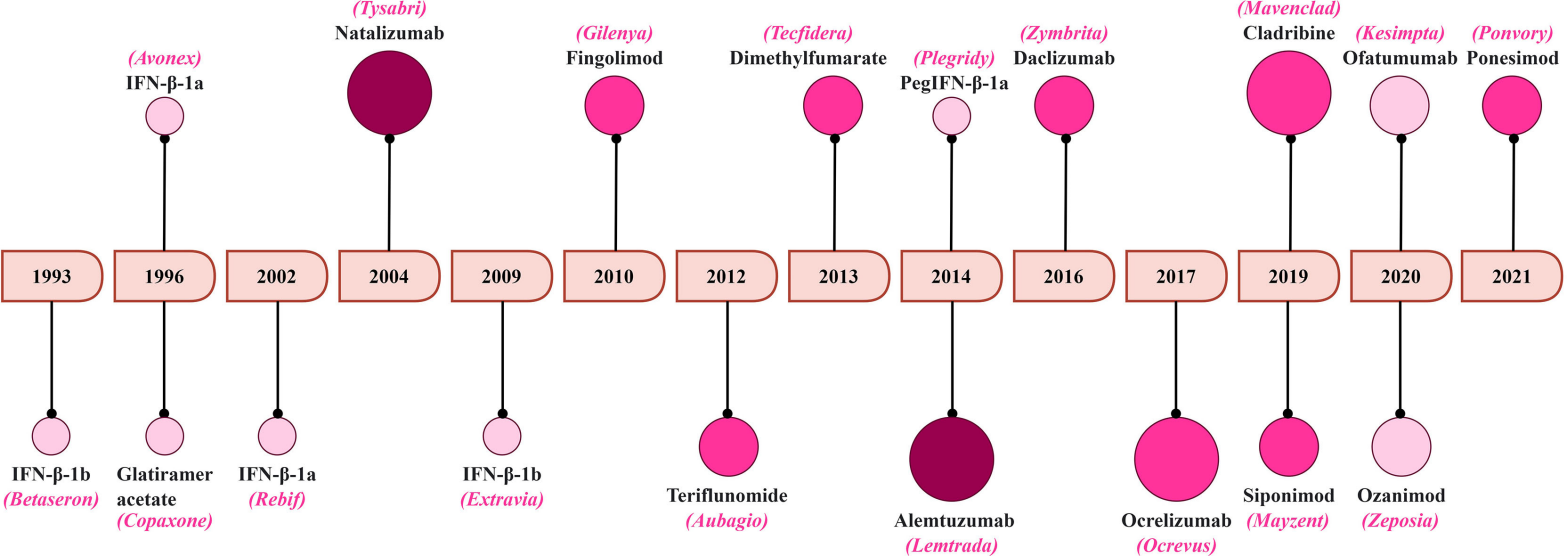
A timeline of FDA approval for MS therapeutics. The increasing color gradient in the circles indicates potential for higher toxicity, while the increasing size of the circles indicates higher efficacy.

MS remains life-lasting disease where its management is aimed to facilitate the recovery after the relapse, delay the disease progression and alleviation of symptoms (222, 248). Here, we summarize the treatments strategies with a focus on T-cell targeting. A schematic representation of the FDA approval for MS therapeutics is summarized in **Figure 5**.

### Disease-modifying treatments (DMTs)

DMTs such as IFN-β and glatiramer acetate, were amongst the first agents commonly prescribed to MS patients (248). Their therapeutic effects were explained by decreasing pro-inflammatory cytokine production, reducing formation of MRI lesions, inhibiting immune cell activation, and lowering matrix metalloproteinase activity (249–251). Three major IFN-β products were approved as the first line MS treatment based on the phase III clinical trial results (252). It was demonstrated that the mechanism of IFN-β therapeutic effect is based on the enhancement of the forkhead box P3 (FoxP3) expression in CD4+ and CD25+ Treg cells (253) and downregulation of HLA expression on antigen presenting cells (254). The side-effects, such as flu-like symptoms, elevated level of hepatic enzymes and injection-site reaction, are mild and could be managed by symptomatic treatment (255). Glatiramer acetate is an alternative to IFN-β, with similar therapeutic effects; however, it has aberrant effects such as dyspnea, palpitation, chest tightness and flushing (256, 257).

### Targeting sphingosine-1-phosphate (S1P) receptor

With our advances in the understanding the lymphocyte role in the pathogenesis of MS, the novel approaches were developed to correct the aberrant immune response and leukocyte infiltration in CNS. The research now focused on the identification of immune-modulating agents and monoclonal antibodies, with the ability to restrain lymphocyte diapedesis into the CNS through the BBB. Addressing this pressing need, the therapeutic potentials of S1P inhibitors in MS were explored. S1P is a sphingolipid that regulates lymphocyte migration and enhances T cell survival (258–260). Fingolimod was the first oral S1P1 modulator approved by the FDA in 2010 for the treatment of MS (248, 251). It is an anti-inflammatory drug with the mechanism of action based on degrading the sphingosine-1-phosphate (S1P) receptor on lymphocytes (250, 251), which is required for their migration to the CNS (248). Phase III clinical trial data indicate higher efficacy of fingolimod in reduction of the relapse rate as compared to IFN-β (261). This drug has multiple side effects including gastroenteritis, back pain, lymphopenia, macular edema and, possibly, bradycardia and atrioventricular blockage. Therefore, it is recommended only for treatment of patients with no prior infection history. Siponimod (also known as BAF312) and ozanimod are also S1P1 modulating agents that displayed therapeutic effects by selectively inhibiting S1P in phase II clinical trials (262, 263). Ponesimod, a selective S1P1 modulator, was tested in double-blind, placebo-controlled trial demonstrating a significant reduction of T1 brain lesions and delayed relapses (264). Later, a phase III clinical trial showed a higher efficacy of ponesimod as compared to teriflunomide on MS activity (265). Ponesimod was well tolerated without safety concerns for MS patients.

### Monoclonal antibody-based therapeutics

A group of monoclonal antibody-based therapeutics were shown to be effective in treatment of MS. One of them is natalizumab, a humanized monoclonal antibody, demonstrated efficacy in Phase III trial by reducing annualized relapse rate, preventing lesion accumulation and decreasing progression of disability (266). The mechanism of therapeutic potency could be explained by prevention of lymphocyte adhesion to VCAM on endothelial cells and α4-integrin receptor on lymphocytes (267). This could block the migration of lymphocytes across the BBB and entering the CNS. Natalizumab also demonstrated better relapse reduction rates and longer-lasting effects as compared to IFN-β (268, 269). It should be noted that natalizumab has limitations such as induction of progressive multifocal leukoencephalopathy, high infusion-related adverse responses and ‘rebounding’ of the diseased-state upon treatment cessation (248, 270, 271).

Since B-cells play a central role in MS pathogenesis, the therapeutic efficacy of monoclonal antibodies targeting this lymphocyte population, such as rituximab, ocrelizumab and ofatumumab, was also assessed in clinical trials. The objective of targeting B-cells is to prevent their diapedesis across the BBB, decrease interaction with T cells as well as restrict pro-inflammatory cytokine production (272). Rituximab and ocrelizumab are widely used monoclonal antibodies, which target B-cell surface antigen CD20 (207, 230). Ocrelizumab selectively depletes B-cells, while preserving the ability for B-cell reconstitution (248). Ofatumumab also inhibits B cell activation and has a similar efficacy as that of ocrelizumab to control this lymphocytes (248, 251). The effectiveness of rituximab and ocrelizumab was demonstrated in phase II trials where decreased number of MRI lesions was found in MS patients (251, 273). Also, the therapeutic potency of ocrelizumab was demonstrated in a phase III trial where ~50% reduction in annual relapse rate was documented in MS (232). Studies have shown that rituximab could be used in patients suffering simultaneously with other autoimmune disorders (274).

Another monoclonal antibody-based therapeutic targeting T and B lymphocytes is alemtuzumab, which binds to CD52 (275). Expression of CD52 was shown on activated T lymphocytes, Tregs and B cells (276–278). It is believed that alemtuzumab depletes CD52 expressing lymphocytes by antibody-dependent cell-mediated cytolysis (ADCC) and complement-dependent cytolysis (CDC) (275). It should be noted that the lymphocyte population gradually becomes reconstituted where B cell counts reach the baseline within 6 months, while T cell counts reach a normal range within 12 months (279, 280). In clinical trials, alemtuzumab has demonstrated higher efficacy in reducing relapses as compared with IFNβ-1α and decreasing confirmed disability worsening (CDW) (281, 282).

### Immune-modulating agents

Additionally, multiple immune-modulating agents are recommended for treatment of MS such as anti-inflammatory agents (283–285), diapedesis inhibitors (286), inhibitors of myelin degradation (249, 253), inhibitors of pro-inflammatory cytokines production (287, 288). Each of these therapeutics, however, have limitations or side effects. It was demonstrated that laquinimod could cause cardiovascular complications (289). Also, mitoxantrone was linked to the development of acute leukemia and hematologic side-effects (268, 290). Hepatotoxicity and teratogenic effects are shown as complications for teriflunomide therapy (248, 290), while hepatotoxicity and progressive multifocal leukoencephalopathy were described in patients treated with DMF (291).

### Myelin-reactive T cell vaccination (TCV)

A novel approach focused on targeting the autologous myelin-reactive T-cells vaccine (292) and administration of autologous, myelin-reactive thymic and Tr1 Tregs was tested in MS (293). Early TCV trials reported decreased counts of the pathogenic, MBP-specific T-cells, which was combined with low toxicity (294). However, failure to meet the primary and secondary study endpoints was reported in phase II clinical study using TCV in MS (295). In 2002, an open label study pointed at a need for multiple TCV doses to prevent relapse post first vaccination. Encouraging results were reported in 2004 during a TCV trial using MBP and MOG activated T-cells in 20 patients, having low therapeutic efficacy of the standard treatment options (294). In this study, the therapeutic effect was determined by the reduction of lesions count, relapse rate and neurological disability. Similar results were reported in phase I/II clinical trial with TCV in 19 subjects where reduced frequency of the relapse without side-effects was reported (296).

Some studying conducted in the last decade also confirmed safety and good tolerability of TCV. In 2012, Karussis et al. investigated the TCV vaccination efficacy in a blinded clinical trial where RPMS patients received multiple injections of attenuated T-cells reactive to 9 different myelin peptides (175). This study demonstrated TCV safety without serious adverse events. In 2016, Seledtsova et al. conducted a study by immunizing 39 patients with PPMS using multiple autologous polyclonal TCV (297). None of the vaccine-treated patients experienced any significant side effect during the entire follow-up period. The results suggest that polyclonal TCV is safe to use, able to induce measurable, long-lasting, anti-inflammatory immune effects in patients with advanced MS. Although TCV has demonstrated safety and limited side effects, still this information is coming from studying using small, selected groups of patients. Only single phase I/II clinical trial was conducted so far, while there was no phase III clinical trial completed. Therefore, the therapeutic efficacy of TCV remains under the investigation.

### Targeting Tregs

One of the novel approaches is based on targeting the lymphocyte populations shown as contributing to MS pathogenesis. One of these lymphocyte population is Tregs, known to control the expansion of auto-aggressive lymphocytes (298). Supporting the potential of these cells are therapeutic target was the effective relapse prevention, even when Tregs were administered post the onset of EAE (299). In another study, nitric oxide-induced CD4+CD25+FoxP3- Tregs were shown to suppress the migration of immune cells through BBB, decreased proliferation of Th17 and lowered secretion of IL-17 in EAE mice model (300). Recently, adoptive cell transfer therapy using engineered Tregs, expressing MOG specific-TCR, was used for treatment of EAE mice (301). Authors reported a positive outcome at both, the initial and the peak stages, in EAE. In another recent study, a phase I trial using CD4+CD25highCD127-FoxP3+ Tregs reported no adverse effects such as relapse of MS, enlarging T2 lesions or visual impairment progression, upon intrathecal administration (110). These reports support the potential of Tregs based cell-therapy in MS, though, further validation in large size cohorts MS patients is required.

There were several reports of the therapeutic potency of engineering antigen-specific Tregs such as a chimeric antigen receptor T cells or CAR-T cells, in MS. Fransson et al. used CD4+ T cells, expressing MOG-targeting CAR and FoxP3, to start treating EAE mice at the height of the clinical symptoms (302). Authors have reported an effective suppression of EAE symptoms which was coupled with reduced IL-12 and IFN-γ production. Also, repeated attempts to induce EAE failed to produce symptoms in treated mice, indicating development of a prolonged protection by Tregs (302). In another study, human Tregs were transduced with MBP-specific TCR and used for treatment of EAE (303). The transduced cells ameliorated EAE symptoms and upregulated the expression of several Tregs markers such as FoxP3, latency-associated peptide and Helios (303). The limiting factor of Treg treatment is that the engineering target-specific Tregs is challenging as there is little known about MS-specific autoantigens (304). Also, individual epitope variation could lead to changes in the target antigens, further compounding the engineering process (305).

## Mechanism of action of drugs used for MS treatments

Our understanding of the therapeutic mechanisms of drugs used for MS treatments remains largely unknown.

### Mechanisms of DMT drugs

It was suggested that IFN-β could increase the production of anti-inflammatory cytokines, inhibit the secretion of pro-inflammatory IL-17 as well as reduce the migration of leukocytes across BBB (306). Following IFN-β administration, increased expression of Th2 (IL-10 and IL-4) and reduced Th1 (IFN-γ and TNF-α) cytokines in CD4+ T cells of MS patients were reported (307). Also, direct interaction between IFN-β and CD4+ T cells may result in regulation of IL-17 expression *via* type I IFN receptor-mediated activation of (STAT)-1 signaling pathway and suppression of STAT3 activity (308) Further, IFN-β could suppress IL-17 secretion by T cells *via* IFN-α/β receptor signaling (309). Additionally, IFN-β was shown to reduce the level of ICAM-1, which is essential for T cells binding to endothelial cells and crossing BBB (310).

The mechanism of Glatiramer acetate, another DMT drug, the therapeutic effect could also target the Th17 cell population by inhibiting the production of IL-17, IFN-γ and IL-6, cytokines required for differentiation of this subset of lymphocytes (311). Glatiramer acetate could promote the development of anti-inflammatory T cells, T-helper 2, and regulatory T cells (312, 313). It was suggested that these Th2 cells could be accumulated in the CNS, producing anti-inflammatory cytokines and reducing the encephalitic effect of pathogenic T cells (313). Glatiramer acetate has been shown to activate Foxp3 which promotes the development of CD4+CD25+ Tregs (311).

### Drugs targeting S1P and S5P receptors

The therapeutic effect of phosphorylated fingolimod is based on its ability to bind to the S1P receptor (subtypes 1,3,4 and 5) on astrocytes, oligodendrocytes, neurons, microglia, and BBB (314). This interaction induces irreversible internalization and degradation of the receptor, which results in lymphocyte sequestration to the secondary lymphoid organs (315). This results in the reduction of peripheral blood lymphocyte count, especially of T cells with naive and memory phenotypes (315). The therapeutic effect of siponimod is similar to that of phosphorylated fingolimod (316). The main differences are that siponimod activity does not require phosphorylation and it mainly targets the SIP receptor subtype 5 (317). Siponimod has neuroprotective activity by increasing oligodendrocyte precursors maturation, limiting astrogliosis, and promoting remyelination (316). Another drug, ozanimod, has similar mechanisms as phosphorylated fingolimod and siponimod by targeting S1P receptor (318).Ozanimod can bind to both, S1P-1 and SIP-5 subtype receptors, reducing peripheral T cell counts and their migration across the BBB (318).

### Monocolonal antibody based therapeutics

Natalizumab administration decreases leukocyte migration into the CNS (319) by blocking the interaction between VCAM-1 on endothelial cells and α4β1-integrin on lymphocytes (320).Another monoclonal antibody-based drug, rituximab, could induce the apoptotic death of B and T cells, especially those with pro-inflammatory CD3+CD20+ phenotype (321). Rituximab could also interact with a core epitope on the extracellular CD20 loop (321) inducing apoptosis *via* ADCC and complement-dependent cytotoxicity mechanisms (322). Natalizumab’s and rituximab’s ability to decrease B cell counts could also contribute to their therapeutic effect on MS (323). As compared to rituximab, ocrelizumab appears to have lower ability to produce an immune response combined with higher therapeutic efficacy (199, 324) This monoclonal antibody-based drug produces a lower level of neutralizing autoantibodies combined with more ADCC complement-dependent cytotoxicity, as compared to rituximab (324).On the downside, ocrelizumab administration could have higher infusion-related reactions (324). Reduction of B cells and CD20+ T combined with increased counts of Tregs was demonstrated when ofatumumab is used for the treatment of MS (325). Additionally, ofatumumab was shown to limit CD4+ T cells secretion of IFN-γ, TNFα, and GM-CSF, as well as a decrease in Th17.1 cell counts (325).

### Immune-modulating agents

The active metabolite of DMF, monomethyl fumarate (MMF) (326), was shown to decrease peripheral blood mononuclear cell counts by inducing apoptosis. The reduced Th1 (IFN-γ and TNF-α) and increased Th2 (IL-4 and IL-5) cytokines production were reported in MS patients treated with DMF (327). MMF could disrupt the expression of genes responsible for the production of adhesion molecules by interfering with the expression of the nuclear factor kappa B transcription factor (328). Additionally, MMF could protect glial cells against oxidative stress leading to significantly reduced axonal damage in MS (327). Intrathecal methotrexate (ITMTX) is hypothesized to reduce astroglial proliferation and scarring (329).

Laquinimod administration impedes Th17 proinflammatory response and reduces the production of pro-inflammatory IL-12 and TNF-α, while promoting secretion of anti-inflammatory IL-4 and IL-10 cytokines (330). These effects were explained by the ability of laquinimod to inhibit NF-κB pathway (331). The migration of macrophages, CD4+, and CD8+ T cells into the CNS was shown to be reduced by laquinimod, by downregulation of the VLA-4 mediated lymphocytes adhesiveness (332). Additionally, decreased axonal damage, synaptic loss, and inflammatory demyelination have been reported in laquinimod’ treated MS (333). Increased serum level of the brain-derived neuroprotective factor was also demonstrated in MS receiving laquinimod, which could contribute to the drug’s neuroprotective efficacy (334).

It was suggested that mitoxantrone could induce apoptosis in B cells, reduce the secretion of pro-inflammatory cytokines, and suppress T cells (335, 336).The mechanism of the therapeutic effect of teriflunomide could include the inhibition of the dihydro-orotate dehydrogenase enzyme required for *de novo* pyrimidine synthesis in lymphocytes (337). This reduced intracellular level of pyrimidine could have a cytostatic effect on B and T cells, consequently reducing their counts in circulation (337). Cladribine is also suggested to affect the B and T cells, as the absolute lymphocyte counts decreased rapidly upon treatment (288). The phosphorylated cladribine was shown to disrupt DNA synthesis by inhibiting enzymes involved in the cell cycle, which was proposed as a mechanism of B and T cell depletion (338)

### TCV treatment

TCV therapeutic efficacy is based on the activation of anti-idiotypic and anti-ergotypic immune mechanisms, by administration of attenuated, self-activated, myelin-reactive T cells (339). This could lead to a direct depletion of pathogenic Th1 cells, as well as activate CD4+ Th2 cells that produce anti-inflammatory cytokines IL-4 and IL-10. Elevated levels of FoxP3+ Tregs, γδT and NK cells were reported when using TCV (340–343).

## Conclusion

Disruption of myelin sheath integrity causes the neuron damage commonly found in CNS tissues from MS. Damage of the laminated structure of myelin sheath covering neuron axon and cells body is believed to be primarily done by Th1 and Th17 lymphocytes. These lymphocytes could produce inflammatory cytokines as well activate pathogenic CTL and Th17 damaging myelin. The reduced activity and counts of Th9 and Tregs was also demonstrated in MS. This data suggests the limited control over lymphocytes differentiation could lead to activation of pathogenic Th1 and Th17 cells. In addition to T lymphocytes, B cells producing anti-myelin proteins antibodies were shown in CNS of MS. These B cells could produce pro-inflammatory cytokines sustaining pathogenic inflammatory milieu in CNS. Also, B cells could facilitate antigen presentation required to activate pathogenic Th1 and Th17 lymphocytes.

These data support the notion of targeting T and B lymphocytes for treatment of MS. Monoclonal antibodies inhibiting lymphocyte migration to CNS were shown effective in MS. Also, anti-CD20 antibodies demonstrated therapeutic efficacy which was explained by reduction of B lymphocyte counts in MS. CAR-T approach targeting Tregs has shown the therapeutic potency in EAE model of MS. Additionally, TCV reducing the myelin-reactive T-cell counts was demonstrated as having therapeutic efficacy in MS.

## Author contributions

MB, SK, and AR contributed to conception and design of the study. SD and LZ organized the database. RL wrote the first draft of the manuscript. SD, LZ, SJ, KS, VL, TK, and EM wrote sections of the manuscript. All authors contributed to manuscript revision, read, and approved the submitted version.

## Funding

This work was funded by grant of NSFC (81901663). Also, this work was supported by the Kazan Federal University Strategic Academic Leadership Program (PRIORITY-2030).

## Conflict of Interest

The authors declare that the research was conducted in the absence of any commercial or financial relationships that could be construed as a potential conflict of interest.

## Publisher’s note

All claims expressed in this article are solely those of the authors and do not necessarily represent those of their affiliated organizations, or those of the publisher, the editors and the reviewers. Any product that may be evaluated in this article, or claim that may be made by its manufacturer, is not guaranteed or endorsed by the publisher.
